# Impact of digital health technology-based nutritional interventions on the nutritional status of hemodialysis patients: a systematic review and meta-analysis

**DOI:** 10.3389/fnut.2025.1681161

**Published:** 2025-10-29

**Authors:** Kai Zhang, Nan Zhang, Ruixue Wang, Shuimiao Wei, Cuiping Ni

**Affiliations:** ^1^School of Nursing, China Medical University, Shenyang, Liaoning, China; ^2^Shenyang Orthopedics Hospital, Shenyang, Liaoning, China

**Keywords:** digital health technology, hemodialysis, nutrition, meta-analysis, systematic review

## Abstract

**Background:**

Chronic kidney disease (CKD) imposes a growing global burden, with hemodialysis (HD) patients facing high malnutrition rates (28% ~ 54%). Nutritional management is critical but challenging due to strict dietary restrictions and limited healthcare monitoring. Digital health technologies (DHTs) offer dynamic, personalized interventions, yet their efficacy remains inconsistent. This systematic review and meta-analysis aims to assess the effects of DHT-based nutritional interventions on the nutritional status of hemodialysis patients.

**Methods:**

We systematically searched PubMed, Embase, Cochrane Library, Web of Science, CINAHL, Scopus, CNKI, CBM, WanFang, and VIP databases from their inception to 21 March 2025, to investigate the impact of DHTs-based nutritional interventions on the nutritional status of hemodialysis patients. Outcomes included biochemical parameters, anthropometric measures, and Modified Quantitative Subjective Global Assessment (MQSGA). Risk-of-bias assessment used Cochrane criteria, and meta-analyses employed RevMan 5.4 with random/fixed-effects models.

**Results:**

A total of 23 literatures were included, involving 6 countries and 2,762 hemodialysis patients. DHT interventions improved the following 13 outcome measures: MQSGA, hemoglobin, albumin, prealbumin, phosphorus, potassium, BMI, mid-arm muscle circumference, triceps skinfold thickness, relative increase in body weight (%), weight gain, blood urea nitrogen, and serum creatinine. However, it had no significant effect on transferrin and calcium. The intervention forms are mainly applications and mobile platforms.

**Conclusion:**

Overall, DHT-based nutritional interventions effectively enhance multiple nutritional indicators in HD patients. However, variability in study quality, intervention formats, and regional disparities limits generalizability. Future research should prioritize high-quality, multicenter RCTs to optimize intervention protocols and explore emerging technologies.

**Systematic review registration:**

Identifier PROSPERO: CRD420251023133.

## 1 Introduction

Chronic kidney disease (CKD) is a progressive disease characterized by a gradual decline in renal function, caused by various factors that induce chronic kidney structural and functional impairments, with a history of kidney damage lasting over 3 months. According to the Global Burden of Disease (GBD) study, CKD’s ranking in the list of global mortality causes has been rising steadily, securing the 13th position in 2016, climbing to 12th in 2017, and is projected to become the fifth leading cause of death globally by 2040 (1, 2). End-stage renal disease (ESRD) is the final stage of chronic kidney disease, characterized by toxin accumulation in the body, uremic symptoms, and various complications; patients in this stage often need dialysis (3, 4). Hemodialysis (HD) is a common method of renal replacement therapy for patients with acute and chronic renal failure. It involves periodically diverting blood outside the body via artificial means to remove metabolic waste and excess fluid, followed by the return of the purified blood to the body (5). Most Hemodialysis patients undergo regular long-term dialysis sessions lasting 3.5–4.5 h, three times a week. Globally, approximately 89% of ESRD patients undergo Hemodialysis (6). As of 2013, approximately one million individuals worldwide were undergoing hemodialysis, with the total number of patients receiving dialysis increasing at an annual rate of 10% (7). In China, the number of hemodialysis patients reached 910,000 by 2023 and is anticipated to continue rising significantly (8).

Hemodialysis patients, due to renal dysfunction, suffer from accumulated uremic toxins and insufficient erythropoietin production. Although long-term hemodialysis is life-saving, it is an invasive procedure that can induce chronic inflammation and increase nutritional consumption; additionally, these patients often require adherence to a low-protein diet, which may further contribute to malnutrition (9, 10). Studies indicate that the global malnutrition prevalence in such patients is as high as 28–54%, and in China, the rate of malnutrition in hemodialysis patients is even higher, ranging from 30.0 to 66.7% (11, 12). If hemodialysis patients are chronically malnourished, they may experience various adverse outcomes such as cognitive impairment, frailty, falls, disability, death, and a reduced quality of life (13, 14). Therefore, nutritional management for hemodialysis patients is crucial to prevent these adverse outcomes. At the same time, hemodialysis patients typically do not require constant hospitalization. After dialysis, they often return home, with some even undergoing treatment at home or in community settings. These challenges render continuous monitoring by healthcare professionals impractical. The strict dietary restrictions imposed on these patients make long-term adherence particularly difficult (15). Furthermore, existing nutritional interventions, which are typically guided by standardized protocols, are often challenging to implement rigorously due to significant individual differences among patients, such as age, comorbidities, and duration of dialysis (16).

The World Health Organization defined digital health as the field of knowledge and practice associated with the development and use of digital technologies to improve health in the “Global Strategy on Digital Health 2020–2025” (17). Digital health technologies are those that can remotely access personal health-related information, including electronic health records, telemedicine or telehealth services, robotic technologies, and mobile health technologies supported by smartphones, wearable devices, applications, and various monitoring devices (17). The development of digital health technology provides new strategies for the nutritional management of hemodialysis patients. Compared with traditional methods, it features dynamic assessment, real-time feedback, efficiency, cross-spatiotemporal accessibility, precision, and personalization. In recent years, it has shown advantages in practical applications for hemodialysis patients. However, current research indicates inconsistent effects of digital health technology on the nutritional management of these patients. Some studies suggest it can improve hemoglobin, albumin, and BMI in hemodialysis patients, while others show no significant effects of the intervention (18, 19). Notably, hemoglobin is not only a key indicator of nutritional status but also the core marker for assessing anemia, one of the most common and clinically critical complications of hemodialysis, closely linked to malnutrition. Anemia affects 91.6–98.2% of hemodialysis patients (20), and its control directly impacts patients’ quality of life and prognosis. Thus, clarifying whether digital health technology-based nutritional interventions can effectively improve hemoglobin (and thereby alleviate anemia) is an essential part of confirming their overall value for nutritional management. This study uses meta-analysis to evaluate the effects of this technology on the nutritional intervention of hemodialysis patients. It aims to provide evidence-based support for developing feasible and effective intervention strategies, ultimately reducing malnutrition in hemodialysis patients.

## 2 Methods

We adhered to the Preferred Reporting Items for Systematic Reviews and Meta-Analyses 2020 (PRISMA 2020) guidelines (21).

### 2.1 Literature search

Zhang Kai and Wang Ruixue searched PubMed, Embase, Cochrane Library, Web of Science, CINAHL, Scopus, China National Knowledge Infrastructure (CNKI), China Biology Medicine disc (CBMdisc), WanFang, and Weipu (VIP) databases. The search strategy integrated Medical Subject Headings (MeSH) and free terms and was customized for each database’s unique characteristics. Search terms included “hemodialysis,” “MHD,” “hemodialysis,” “renal dialysis,” “mobile applications,” “smartphone,” “wearable electronic devices,” “digital health,” “telemedicine,” “artificial intelligence,” “internet,” “mhealth,” “web,” “wechat,” “virtual reality,” “VR,” “virtual environment,” “virtual simulation,” “nutrition,” “nutri*,” “supplement*,” “diet therapy,” “diet*,” “dietary.” We conducted a manual search using the literature tracing method to comprehensively supplement the literature. Our search encompassed the period from the establishment of each database to 21 March 2025. The study is registered in PROSPERO: CRD420251023133. For illustration, the specific search strategy is detailed in Supplementary Table S1.

### 2.2 Inclusion and exclusion criteria

#### 2.2.1 Inclusion criteria

Population (P): Patients undergoing hemodialysis and age ≥18 years. Intervention (I): Nutrition interventions delivered through digital health technologies (core components including mobile health, artificial intelligence, telehealth, or virtual reality) specifically designed for HD patients. Comparison (C): Usual care, routine care, conventional care, or standard care without integration of m-health/digital health components. Outcomes (O): Nutritional outcomes included the following: Biochemical parameters (e.g., serum albumin, prealbumin, and phosphorus); anthropometric measurements (e.g., body mass index and mid-arm muscle circumference); scores from internationally recognized nutritional assessment scales (e.g., Modified Quantitative Subjective Global Assessment, MQSGA). Study type (S): Randomized controlled trials (RCTs) reporting means, standard deviations, and explicit sample sizes to allow effect size pooling for primary/secondary outcomes. Language: Studies published in Chinese or English.

#### 2.2.2 Exclusion criteria

(1) Meetings, policy documents, or research proposals. (2) The interventions were not specific and were only follow-up interventions conducted via telephone, email, or WeChat. (3) Repeated publication.

### 2.3 Data extraction

Two researchers (Zhang Kai and Wang Ruixue), who had received training in evidence-based nursing, searched the literature independently. Two researchers independently read the title and abstract for preliminary screening and carefully read the full text. According to the inclusion and exclusion criteria of the literature, the included literature was determined. Two researchers extracted literature information, including the literature author, publication time, country, sample size, age, dialysis age, intervention cycle, carrier format, intervention content, and outcome measures. If there were any differences, the third researcher (Ni Cuiping) was consulted to assist in the judgment.

### 2.4 Risk-of-bias assessments

Three reviewers (Zhang Kai, Wang Ruixue, and Zhang Nan) assessed the quality of the studies according to the Cochrane Collaboration’s tool for risk of bias, which included seven categories of risk of bias: randomized sequence generation, allocation concealment, participant blinding, outcome assessor blinding, incomplete data, selective reporting, and other bias (22). Each item was evaluated as “high risk,” “low risk,” and “unclear.” Disagreements were adjudicated by a fourth reviewer (Ni Cuiping). The methodological quality of the included trials was categorized into three tiers using the following evaluation framework: (1) Low-quality trials were defined as those exhibiting high risk of bias in either randomization procedures or allocation concealment, irrespective of other methodological domains; (2) High-quality trials required both robust randomization and allocation concealment, with all remaining methodological components demonstrating low or unclear risk; (3) Moderate-quality trials encompassed studies that neither satisfied the stringent criteria for high quality nor met the threshold for low-quality classification (23).

### 2.5 Statistical analysis

Data analysis was performed using Review Manager (RevMan) software version 5.4. All continuous outcomes were pooled using Standardized Mean Differences (SMD) with 95% confidence intervals (CI). This approach was chosen because the outcomes encompassed heterogeneous measures, including unitless comprehensive scores (MQSGA), biochemical parameters with differing units (e.g., albumin, phosphorus), and anthropometric measures (e.g., BMI, mid-arm muscle circumference). Although units were standardized for the same indicator where necessary, SMD allows for the comparison of effect magnitudes across these fundamentally different types of outcomes, thereby facilitating an integrated assessment of the intervention’s impact on multidimensional nutritional status. Heterogeneity was evaluated through the Chi-square test and Cochran’s Q statistic. A fixed-effects model was applied when heterogeneity was non-significant (*p* ≥ 0.1 and *I*^2^ < 50%), while a random-effects model was utilized in cases of substantial heterogeneity (*p* < 0.1 or *I*^2^ ≥ 50%). For heterogeneous outcomes (*I*^2^ ≥ 50%), subgroup analyses were conducted to explore potential sources of heterogeneity, stratified by 1. Digital health technology types (application program vs. mobile platform); 2. Intervention duration (≥ 6 months vs. < 6 months). Publication bias was assessed via funnel plot symmetry inspection. Sensitivity analyses were implemented through a leave-one-out approach to verify result robustness. Statistical significance was defined as a two-tailed *p*-value < 0.05.

## 3 Results

### 3.1 Literature screening process and results

**Figure 1:**
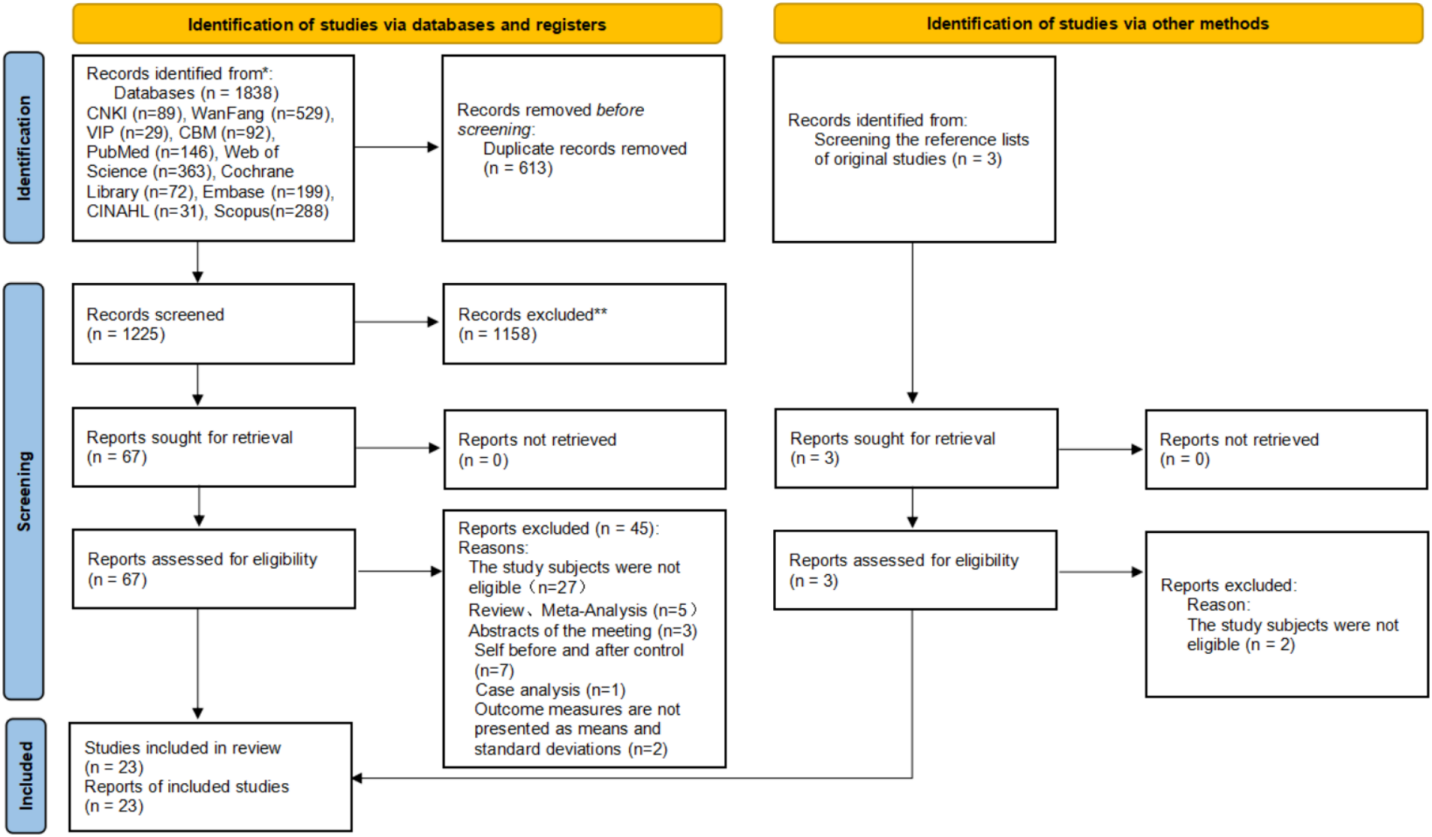
PRISMA diagram of searching and screening process.

A total of 1,838 records were identified from databases and registers, including CNKI (*n* = 89), WanFang (*n* = 529), VIP (*n* = 29), CBM (*n* = 92), PubMed (*n* = 146), Web of Science (*n* = 363), Cochrane Library (*n* = 72), Embase (*n* = 199), CINAHL (*n* = 31), and Scopus (*n* = 288). After removing 613 duplicate records, 1,225 records were screened, and 1,158 records were excluded because they were not relevant to the research topic. Subsequently, 67 reports were sought for retrieval, with none being unretrieved. Following eligibility assessment of these 67 reports, 45 were excluded for the following reasons: the study subjects were not eligible (*n* = 27), reviews or meta-analysis (*n* = 5), meeting abstracts (*n* = 3), self before-and-after control (*n* = 7), case analysis (*n* = 1), and outcome measures not presented as means and standard deviations (*n* = 2). Additionally, 3 records were identified by screening the reference lists of original studies; all 3 reports were retrieved successfully, and after eligibility assessment, 2 records were excluded because the study subjects were not eligible. Finally, a total of 23 studies were included in this review. This study adheres to the PRISMA guidelines, and the literature searching and screening process is outlined in Figure 1.

### 3.2 The basic characteristics of the included literature

A total of 23 studies were included in the analysis, originating from 6 countries: China (*n* = 18) (18, 24–40), South Korea (*n* = 1) (19), Thailand (*n* = 1) (41), Iran (*n* = 1) (42), Malaysia (*n* = 1) (43), and the United States (*n* = 1) (44). In terms of publication timeframe, one study was published between 2011 and 2015 (44), four studies were published between 2016 and 2020 (24, 25, 27, 28), and 18 studies were published between 2021 and 2025 (18, 19, 26, 29–39, 41–43). These studies involved 2,762 hemodialysis patients in total, with 1,382 in the intervention group and 1,380 in the control group. Of the included studies, 19 focused on application-based interventions, including those utilizing WeChat and apps (19, 24–31, 33–35, 37, 38, 40–44). The remaining four studies adopted a mobile platform (18, 32, 36, 39). The basic characteristics of the included literatures are shown in Table 1.

**Table 1:** Basic information of the included literature.

Author, year	Country	Sample size (T/C)	Age (T/C)	Dialysis age (months)	Intervention cycle	Carrier format	Intervention content	Outcome measures
Kong YX, 2017 (24)	China	50/50	45.45 ± 6.52 44.65 ± 6.78	-	3 months	Application program (WeChat)	Conducted dietary education and answered questions via WeChat	Relative increase in body weight (%), Serum albumin, hemoglobin, potassium, and phosphorus
Wang J, 2020 (25)	China	250/250	36.45 ± 12.98 35.12 ± 12.81	-	2 months	Application program (WeChat)	Delivered knowledge on disease and dialysis, provided diet and exercise guidance, and adjusted intervention plans based on nutritional status	Albumin, prealbumin, and transferrin
Fan JZ, 2022 (26)	China	44/45	75.65 ± 10.78 75.45 ± 11.32	68.78 ± 30.25 69.32 ± 31.17	5 months	Application program (WeChat)	Provided nutrition education via WeChat and face-to-face, and developed personalized meal plans	Hemoglobin, potassium, calcium, phosphorus, creatinine, blood urea nitrogen, and transferrin; mid arm muscle circumference, triceps skinfold thickness, and BMI.
Zhang N, 2022 (18)	China	79/79	67.07 ± 13.28 67.53 ± 12.12	36.57 ± 8.96 34.12 ± 8.52	3 months	Mobile platform	Nutrition team conducted a nutritional index assessment, precise nutrition and dry weight management, and provided targeted guidance based on a 3-day diet record analysis	Hemoglobin, prealbumin, albumin, phosphorus, potassium; MQSGA; BMI.
Zeng T, 2020 (27)	China	50/50	51.10 ± 9.57 53.10 ± 9.56	-	6 months	Application program (WeChat, QQ)	Nutrition management team provided psychological counseling, outcome feedback, meal planning, and personalized guidance	MQSGA, Albumin, transferrin, hemoglobin; mid arm muscle circumference, triceps skinfold thickness.
Deng FY, 2019 (28)	China	61/61	-	-	6 months	Application program (WeChat)	The intervention was structured around five phases based on the Timing It Right theory.	Hemoglobin, serum protein, potassium, blood urea nitrogen, and serum creatinine.
Zhang JQ, 2024 (29)	China	50/50	69.14 ± 3.11 69.28 ± 3.19	90.12 ± 13.44 91.68 ± 14.28	3 months	Application program (WeChat)	Conducted family education, provided diet management reports and family peer support, shared experiences, and kept diet diaries via WeChat	Hemoglobin, albumin, prealbumin, and transferrin.
Shi LR, 2023 (30)	China	55/55	56.64 ± 9.39 57.38 ± 9.84	-	3 months	Application program (WeChat)	Provided personalized nutrition guidance and regular nutrition knowledge dissemination, and carried out online interactive sharing and plan assessment-adjustment	Hemoglobin, albumin, triceps skinfold thickness.
Duan F, 2023 (31)	China	74/74	54.79 ± 5.98 55.34 ± 5.21	2.25 ± 0.53 2.27 ± 0.48	2 months	Application program (WeChat)	Provided guidance via educational videos and personalized diet-lifestyle plans, and conducted dietary counseling and supervision via WeChat	Albumin, prealbumin; MQSGA
Shi SH, 2021 (32)	China	79/79	50.68 ± 11.09 49.24 ± 12.68	57.81 ± 31.80 56.37 ± 33.44	3 months	Mobile platform	Provided comprehensive personalized dietary guidance and dietary tips, and implemented intervention via educational animation videos, doctor-patient video interaction, and patient experience-sharing sessions	MQSGA, serum albumin, hemoglobin, phosphorus, calcium, potassium, serum creatinine, and blood urea nitrogen.
Dong XY, 2022 (33)	China	74/74	53.22 ± 10.07 54.12 ± 9.51	56.11 ± 13.08 61.77 ± 18.89	6 months	Application program (WeChat)	Conducted comprehensive dietary education: 6–18 videos; monitoring for unhealthy habits; food discussions; patient peer exchange; video Q&A sessions	Hemoglobin, albumin, and prealbumin
Pack S, 2021 (19)	South Korea	37/38	52.00 ± 10.01 50.66 ± 9.15	-	8 weeks	Application program	Carried out dietary self-management in 3 phases: introduction, implementation, and maintenance	Phosphorus, potassium, and albumin.
Wang LJ, 2024 (34)	China	110/110	-	-	3 months	Application program (WeChat)	Nutrition team conducted education via online consultation groups, needs assessment surveys, and PPT/video popularization, and adjusted diet via a food nutrient APP	Potassium, phosphorus, mid arm muscle circumference, albumin, prealbumin, transferrin, creatinine, and blood urea nitrogen, MQSGA
Teong LF, 2022 (43)	Malaysia	33/33	47.5 ± 15.3 49.15 ± 13.63	-	3 months	Application program (phosphate mobile app, MyKidneyDiet-Phosphate Tracker)	Provided dietary guidance via 6 animated educational videos and calculated personalized dietary intake	Phosphorus, calcium
Wu LF, 2021 (35)	China	51/51	44.90 ± 10.02 45.43 ± 10.70	49.88 ± 23.01 51.27 ± 18.54	6 months	Application program (App)	The APP calculated recommended intake and monitored actual consumption, adjusted in a timely manner based on comparison, and provided food database queries	MQSGA, Relative increase in body weight (%), body Weight gain, albumin, hemoglobin, and mid-arm muscle circumference.
Thongsunti A, 2024 (41)	Thailand	40/40	-	-	6 months	Application program (LINE application)	Watched 3 dietary education videos and received personalized dietary guidance and supervision	Phosphorus
Torabikhah M, 2023 (42)	Iran	35/35	45.2 ± 1.4 46.7 ± 1.5	38.4 ± 2.4 40.8 ± 2.4	3 months	Application program (Di Care app)	Covered 7 topics via videos: the importance of HD, management of HD complications, diet, fluid intake restriction, physical activity, vascular access care, and medications	Potassium, phosphorus, albumin, ferritin, body weight gain
Zhou QQ, 2024 (36)	China	30/30	55.08 ± 10.72 54.58 ± 10.27	108.36 ± 26.4 107.4 ± 25.8	2 months	Mobile platform	Carried out online consultation, doctor-patient interaction, health education and assessment, and developed precise nutrition and dry weight plans	Prealbumin, phosphorus.
Wei DM, 2024 (37)	China	30/30	45.91 ± 7.53 46.72 ± 7.48	37.12 ± 10.33 36.98 ± 10.26	3 months	Application program (WeChat)	Nutrition team provided dietary guidance via articles/videos, conducted personalized nutrition assessment and counseling, and organized online educational lectures, Q&A, and diet log supervision	MQSGA, calcium and phosphorus
Welch JL, 2013 (44)	The United States	24/20	53.0 ± 15.1 47.1 ± 11.5		6 weeks	Application program (DIMA)	Recorded food and beverage intake, and provided assessment and feedback based on the entered data to meet the prescribed nutritional intake	Body weight gain
Zhu XB, 2021 (38)	China	43/43	41.2 ± 14.9 40.4 ± 14. 8	141.6 ± 54 135.6 ± 55.2	6 months	Application program (WeChat)	Conducted one-on-one health education sessions every 3 weeks and developed personalized dietary plans	Albumin, hemoglobin
Li HT, 2024 (39)	China	40/40	52.39 ± 6.53 50.14 ± 6.21	37.68 ± 5.76 36.64 ± 6.53	6 months	Mobile platform	Provided personalized plans and digital dietary education, conducted monitoring and interactive Q&A via the platform, and adjusted plans based on regular re-assessments	Phosphorus, calcium, albumin, and prealbumin
Yao DD, 2023 (40)	China	43/43	44.53 ± 4.41 44.38 ± 4.37	13.59 ± 3.61 13.38 ± 3.57	3 months	Application program (WeChat)	Developed personalized low-phosphorus (<800 mg/day) and high-protein meal plans based on 3-day diet records (considering body fat, disease status, and calcium-phosphorus metabolism), and provided guidance on food selection and portion	Calcium, phosphorus

MQGSA, Modified Quantitative Subjective Global Assessment; BMI, Body Mass Index; HD, hemodialysis; Q&A, Question and Answer; -, no report.

### 3.3 The results of the risk of bias assessments

In total, 13 studies were assessed as having low risk of bias in randomized sequence generation (18, 25–29, 32, 39–44); 4 studies were rated as high risk due to the use of pseudo-randomization (24, 31, 34, 35); 6 studies, although stating the use of randomization, did not specify the type of randomization method used and were therefore rated as unclear (19, 30, 33, 36–38). Three studies reported allocation concealment and were rated as low risk (26, 32, 41); the remaining 20 studies were rated as unclear (18, 19, 24, 25, 27–31, 33–40, 42–44). Blind outcome assessment is challenging due to the unique nature of nutritional interventions, and 22 studies did not report blind outcome assessment, leading to unclear ratings (18, 19, 24–31, 33–44); one study reported blind outcome assessment, leading to low risk (32). Since the outcome measures in this study are mostly laboratory test results, which are relatively objective, the lack of blinding of outcome assessors has minimal impact on the results, and thus all 23 studies were rated as low risk (18, 19, 24–44). A total of 16 studies had complete outcome data and were rated as low risk (18, 24, 25, 27, 29–31, 33, 36–43); 7 studies reported reasons for dropout but did not describe whether an intention-to-treat analysis was conducted, resulting in an unclear rating (19, 26, 28, 32, 34, 35, 44). In total, 4 studies did not exhibit selective reporting and were rated as low risk (19, 41–43); 19 studies, although reporting both positive and negative results, did not report whether the RCT was registered, leading to an unclear rating (18, 24–40, 44). Three studies had no other biases and were rated as low risk (26, 28, 32); the remaining 20 studies did not report on quality control measures and were rated as unclear (18, 19, 24, 25, 27, 29–31, 33–44). The four studies were evaluated as low quality (24, 31, 34, 35), three studies as high quality (26, 32, 41), and the remaining 16 studies as medium quality (18, 19, 25, 27–30, 33, 36–40, 42–44). The figure of the risk of bias is shown in Supplementary Figure S1, while a summary of the risk of bias is shown in Supplementary Figure S2.

### 3.4 Meta-analysis results

#### 3.4.1 Main outcome measures

##### 3.4.1.1 MQSGA

Seven studies reported the MQSGA score (18, 27, 31, 32, 34, 35, 37). The meta-analysis showed a significant pooled effect on the MQSGA score (SMD = −1.48, 95% CI: −1.93 to −1.03). Considerable heterogeneity was observed (*I*^2^ = 95%, *p* < 0.001). The forest diagram is shown in Supplementary Figure S3.

#### 3.4.2 Biochemical indicators

##### 3.4.2.1 Hemoglobin

A total of 11 studies reported hemoglobin levels (18, 24, 26–30, 32, 33, 35, 38). The meta-analysis showed a significant pooled effect on the hemoglobin (SMD = 0.86, 95% CI: 0.67 to 1.05). Considerable heterogeneity was observed (*I*^2^ = 80%, *p* < 0.001). The forest diagram is shown in Supplementary Figure S4.

##### 3.4.2.2 Serum albumin

A total of 18 studies reported serum albumin levels (18, 19, 24–35, 38, 39, 42, 43). The meta-analysis showed a significant pooled effect on albumin (SMD = 0.81, 95% CI: 0.63 to 0.99). Significant heterogeneity was observed (*I*^2^ = 88%, *p* < 0.001). The forest diagram is shown in Supplementary Figure S5.

##### 3.4.2.3 Prealbumin

Seven studies reported prealbumin levels (18, 25, 29, 31, 33, 36, 39). The meta-analysis revealed a significant pooled effect on prealbumin (SMD = 0.60, 95% CI: 0.48 to 0.72). Heterogeneity was observed (*I*^2^ = 41%, *P* < 0.001). The forest diagram is shown in Supplementary Figure S6.

##### 3.4.2.4 Transferrin

Five studies reported serum transferrin levels (25–27, 29, 34). The meta-analysis showed no significant pooled effect on transferrin (SMD = 0.14, 95% CI: −0.84 to 1.11). Heterogeneity was substantial (*I*^2^ = 98%, *p* = 0.78). The forest diagram is shown in Supplementary Figure S7.

##### 3.4.2.5 Calcium

Seven studies reported serum calcium levels (26, 32, 34, 37, 39, 40, 43). The meta-analysis showed no significant pooled effect on calcium (SMD = −0.04, 95% CI: −0.65 to 0.58). Considerable heterogeneity was observed (*I*^2^ = 94%, *p* = 0.91). The forest diagram is shown in Supplementary Figure S8.

##### 3.4.2.6 Phosphorus

Thirteen studies reported serum phosphorus levels (18, 19, 24, 26, 32, 34, 36, 37, 39–43). The meta-analysis showed a significant pooled effect on phosphorus (SMD = −0.71, 95% CI: −0.97 to −0.46). Considerable heterogeneity was observed (*I*^2^ = 90%, *p* < 0.001). The forest diagram is shown in Supplementary Figure S9.

##### 3.4.2.7 Potassium

In total, 8 studies reported serum potassium levels (18, 19, 24, 26, 28, 32, 34, 42). The pooled effect size was statistically significant (SMD = − 0.67, 95% CI: −1.19 to −0.15). A high degree of heterogeneity was observed (*I*^2^ = 93%, *p* = 0.01). The forest diagram is shown in Supplementary Figure S10.

#### 3.4.3 Anthropometric indicators

##### 3.4.3.1 Relative increase in body weight (%)

In total, 2 studies reported the relative increase in body weight (%) (24, 35). The meta-analysis showed a statistically significant effect (SMD = −1.00, 95% CI: −1.72 to −0.28). Heterogeneity was substantial (*I*^2^ = 83%, *p* = 0.007). The forest diagram is shown in Supplementary Figure S11.

##### 3.4.3.2 Body weight gain

In total, 2 studies reported body weight gain (42, 44). The meta-analysis showed a significant pooled effect (SMD = − 0.71, 95% CI: −1.12 to −0.31). Moderate heterogeneity was observed (*I*^2^ = 44%, *p* < 0.001). The forest diagram is shown in Supplementary Figure S12.

##### 3.4.3.3 BMI

In total, 2 studies reported body mass index (BMI) (18, 26). The meta-analysis showed a significant pooled effect (SMD = 0.31, 95% CI: 0.06 to 0.56). Moderate heterogeneity was observed (*I*^2^ = 37%, *p* = 0.02). The forest diagram is shown in Supplementary Figure S13.

##### 3.4.3.4 Mid-arm muscle circumference

In total, 4 studies reported mid-arm muscle circumference (26, 27, 34, 35)^.^ The meta-analysis showed a significant effect (SMD = 1.92, 95% CI: 0.46 to 3.38). High heterogeneity was observed (*I*^2^ = 98%, *p* = 0.01). The forest diagram is shown in Supplementary Figure S14.

##### 3.4.3.5 Triceps skinfold thickness

In total, 2 studies reported triceps skinfold thickness (26, 27). The meta-analysis showed a significant effect (SMD = 1.16, 95% CI: 0.13 to 2.18). Considerable heterogeneity was observed (*I*^2^ = 91%, *p* = 0.03). The forest diagram is shown in Supplementary Figure S15.

#### 3.4.4 Secondary outcome indicators

##### 3.4.4.1 Urea nitrogen

In total, 3 studies reported blood urea nitrogen levels (26, 28, 32). The meta-analysis showed a significant effect (SMD = −0.27, 95% CI: −0.48 to −0.06). No heterogeneity was observed (*I*^2^ = 0%, *p* = 0.01). The forest diagram is shown in Supplementary Figure S16.

##### 3.4.4.2 Serum creatinine

In total, 3 studies reported serum creatinine levels (26, 28, 32). The meta-analysis showed a significant pooled effect (SMD = −0.80, 95% CI: −1.23 to −0.38). Considerable heterogeneity was observed (*I*^2^ = 74%, *p* < 0.01). The forest diagram is shown in Supplementary Figure S17.

### 3.5 Publication bias

For the outcomes with ≥ 10 included studies, funnel plots were used to assess publication bias for hemoglobin, albumin, and phosphorus. The funnel plots for hemoglobin and albumin showed slight asymmetry, indicating possible publication bias. This might be due to small sample sizes, low study quality, design differences, or reporting standardization in some included studies. The funnel plot for phosphorus was largely symmetric, suggesting minimal publication bias. The funnel plots are shown in Supplementary Figures S18–S20.

### 3.6 Sensitivity analyses

Sensitivity analyses were performed on outcome indicators with ≥ 3 studies by sequentially removing individual studies. For transferrin, after excluding a study by Wang LJ (34), the pooled effect size became statistically significant (*p* < 0.001). For mid-arm muscle circumference, excluding a study by Zeng T made the result non-significant (*p* = 0.08) (27). Similarly, for blood urea nitrogen and serum creatinine, removing studies by Fan JZ (26) and Shi SH (32), respectively, also led to non-significant results (*p* = 0.09 and *p =* 0.05). These four indicators showed unstable results. In contrast, results for the other indicators remained stable after sensitivity analysis. The differential sensitivity analyses are presented in Table 2.

**Table 2:** Sensitivity analysis of the variable results with differences.

Variable	Trials	Participants	Total	SMD (95% CI)	*p-*value
Transferrin	5	504	1,009	0.14 (−0.84, 1.11)	0.78
After removing Wang LJ, 2024 (34)	4	394	789	0.58 (0.39, 0.78)	<0.001
Serum creatinine	3	182	363	−0.80 (−1.23, −0.38)	<0.001
After removing Shi SH, 2021 (32)	2	103	206	−0.78 (−1.15, −0.00)	0.05
Urea nitrogen	3	182	363	−0.27 (−0.48, −0.06)	0.01
After removing Fan JZ, 2022 (26)	2	138	274	−0.21 (−0.44, 0.03)	0.09
Mid-arm muscle circumference	4	255	511	1.92 (0.46, 3.38)	0.01
After removing Zeng T, 2020 (27)	3	205	411	1.55 (−0.20, 3.29)	0.08

In this table, “Participants” refers to the number of participants in the intervention group, and “Total” refers to the total number of participants (including both intervention and control groups).

### 3.7 Subgroup analysis

#### 3.7.1 MQSGA

Intervention duration: For ≥ 6 months, 202 patients, SMD = −3.06 (95% CI: −3.54 to −2.57, *p* < 0.001) (27, 35); for < 6 months, 743 patients, SMD = −0.84 (95% CI: −1.18 to −0.50, *p* < 0.001) (18, 31, 32, 34, 37). Digital health technology types: application program-based nutrition intervention involving 630 patients, SMD = -1.92 (95% CI: -2.81 to -1.03, *p* < 0.001); mobile platform-based nutrition intervention in 315 patients, SMD = -0.46 (95% CI: -0.68 to -0.24, *p* < 0.001) (18, 32). The subgroup analysis is shown in Table 3 and Supplementary Figure S3.

**Table 3:** The results of subgroup analysis of each outcome indicator.

Variable	Trials	Participants	Total	SMD (95% CI)	*p* value
MQSGA					
≥ 6 months	2	101	202	-3.06 (-3.54, -2.57)	< 0.001
<6 months	5	372	743	-0.84 (-1.18, -0.50)	< 0.001
Application program	5	315	630	-1.92 (-2.81, -1.03)	< 0.001
Mobile platform	2	158	315	-0.46 (-0.68, -0.24)	< 0.001
Hemoglobin					
≥ 6 months	5	240	479	1.02 (0.53, 1.51)	< 0.001
<6 months	6	340	678	0.73 (0.41, 1.05)	< 0.001
Application program	9	422	842	0.99 (0.69, 1.28)	< 0.001
Mobile platform	2	158	315	0.36 (0.13, 0.58)	0.002
Albumin					
≥ 6 months	6	280	559	0.94 (0.51, 1.38)	< 0.001
<6 months	12	879	1757	0.74 (0.41, 1.08)	< 0.001
Application program	15	978	1957	0.87 (0.56, 1.18)	< 0.001
Mobile platform	3	198	395	0.51 (0.23, 0.79)	< 0.001
Calcium					
Application program	5	260	521	0.22 (-0.47, 0.90)	0.54
Mobile platform	2	119	237	-0.65 (-1.44, 0.14)	0.11
Phosphorus					
≥6 months	2	80	160	-1.12 (-2.62, 0.39)	0.15
<6 months	11	570	1141	-0.65 (-1.03, -0.26)	0.001
Application program	9	422	846	-0.76 (-1.17, -0.35)	< 0.001
Mobile platform	4	228	455	-0.62 (-1.38, 0.14)	0.11
Potassium					
Application program	6	335	671	-0.78 (-1.47, -0.09)	0.03
Mobile platform	2	158	315	-0.34 (-0.56, -0.11)	0.003

In this table, “Participants” refers to the number of participants in the intervention group, and “Total” refers to the total number of participants (including both intervention and control groups).

#### 3.7.2 Hemoglobin

Intervention duration: For ≥ 6 months of intervention involving 479 patients, the SMD = 1.02 (95% CI: 0.53 to 1.51, *p* < 0.001) (27, 28, 33, 35, 38); For < 6 months of intervention involving 678 patients, the SMD = 0.73 (95% CI: 0.41 to 1.05, *p* < 0.001) (18, 24, 26, 29, 30, 32). Digital health technology types: For application program-based nutritional interventions across 842 patients, the SMD = 0.99 (95% CI: 0.69 to 1.28, *p* < 0.001) (24, 26–30, 33, 35, 38); for mobile platform-based nutritional interventions across 315 patients, the SMD = 0.36 (95% CI: 0.13 to 0.58, *p* = 0.002) (18, 32). The subgroup analysis is shown in Table 3 and Supplementary Figure S4.

#### 3.7.3 Albumin

Intervention duration: For ≥ 6 months, 559 patients, SMD = 0.94 (95% CI: 0.51 to 1.38), *p* < 0.001 (27, 28, 33, 35, 38, 39); for < 6 months, 1757 patients, SMD = 0.74 (95% CI: 0.41 to 1.08), *p* < 0.001 (18, 19, 24–26, 29–32, 34, 42, 43). Digital health technology types: application program-based nutrition intervention, 1957 patients, SMD = 0.87 (95% CI: 0.56 to 1.18), *p* < 0.001; mobile platform-based nutrition intervention, 395 patients, SMD = 0.51 (95% CI: 0.23 to 0.79), *p* < 0.001 (18, 32, 39). The subgroup analysis is shown in Table 3 and Supplementary Figure S5.

#### 3.7.4 Calcium

Digital health technology types: application program-based nutrition intervention, 521 patients, SMD = 0.22 (95% CI: −0.47 to 0.90), *p* = 0.54 (26, 34, 37, 40, 43); mobile platform-based nutrition intervention, 237 patients, SMD = −0.65 (95% CI: −1.44 to 0.14), *p* = 0.11 (32, 39). The subgroup analysis is shown in Table 3 and Supplementary Figure S8.

#### 3.7.5 Phosphorus

Intervention duration: For ≥ 6 months, 160 patients, SMD = -1.12 (95% CI: −2.62 to 0.39), *p* = 0.15; for < 6 months, 1141 patients, SMD = -0.65 (95% CI: −1.03 to −0.26), *p* = 0.001 (18, 19, 24, 26, 32, 34, 36, 37, 40, 42, 43). Digital health technology types: application program-based nutrition intervention, 846 patients, SMD = −0.76 (95% CI: −1.17 to −0.35), *p* < 0.001 (19, 24, 26, 34, 37, 40–43); mobile platform-based nutrition intervention, 455 patients, SMD = −0.62 (95% CI: −1.38 to 0.14) (18, 32, 36, 39), *p* = 0.11. The subgroup analysis is shown in Table 3 and Supplementary Figure S9.

#### 3.7.6 Potassium

Digital health technology types: application program-based nutrition intervention, 671 patients, SMD = −0.78 (95% CI: −1.47 to −0.09), *p* = 0.03 (19, 24, 26, 28, 34, 42); mobile platform-based nutrition intervention, 315 patients, SMD = −0.34 (95% CI: −0.56 to −0.11), *p* = 0.003 (18, 32). The subgroup analysis is shown in Table 3 and Supplementary Figure S10.

For other outcome indicators, subgroup analysis could not be conducted due to the small number of included studies.

## 4 Discussion

A total of 23 studies were included, of which four were low quality, three high quality, and 16 moderate quality. Overall, quality was acceptable but not high. The overall meta-analysis indicated that digital health technology-based nutrition interventions had no significant effects on transferrin and calcium but had significant effects on hemoglobin, albumin, prealbumin, phosphorus, potassium, MQSGA score, relative increase in body weight (%), weight gain, BMI, mid-arm muscle circumference, triceps skinfold thickness, blood urea nitrogen, and serum creatinine. This suggests these interventions may improve nutrition in hemodialysis patients. To further explore potential heterogeneity in the results, subgroup analyses were conducted on six outcomes: hemoglobin, albumin, calcium, potassium, phosphorus, and MQSGA. For phosphorus, subgroup analyses found no significant difference in the ≥ 6-month intervention subgroup and no significant effect of mobile platform-based interventions, inconsistent with the overall meta-analysis. However, regarding this result, since only 2 studies were included in the subgroup analysis, the strength of evidence is unstable. Thus, this result should be interpreted with caution, and more follow-up studies with a duration of ≥ 6 months should be conducted in the future. Other subgroup analyses were generally in line with the overall results. Additionally, sensitivity analyses showed unstable results for transferrin, serum creatinine, blood urea nitrogen, and mid-arm muscle circumference. Caution is needed in interpreting the findings. More high-quality studies are required to validate these results.

Turning to the specific methods employed, the included studies primarily employed the following approaches: 1. Application programs: Mobile applications (Apps): Customized features such as dietary logging, nutrient calculation, and real-time feedback were used to deliver personalized guidance. For instance, Teong et al. (43) developed a hyperphosphatemia management app integrated with a food database and phosphorus intake tracker, enabling patients to generate daily phosphorus reports and receive adjustment recommendations. Similarly, Wu et al. (35) combined IoT-enabled smart water bottles and scales to monitor fluid and nutrient intake in real time, using algorithms to compare actual values with recommended targets and dynamically optimize dietary plans. Social media platforms (e.g., WeChat): These were utilized for health education, interactive consultations, and peer support. For example, Kong et al. (24) disseminated low-protein diet educational videos via WeChat and established patient groups for recipe sharing and management experience exchanges. Additionally, Wang et al. (34) employed a WeChat mini-program (“Food Nutrient Handbook”) to provide daily meal recommendations and dynamically assess patient needs through online questionnaires. 2. Mobile platforms: Remote monitoring and online education: Shi et al. (32) implemented an “Internet Plus” nutrition education model, uploading animated tutorials, hosting virtual classrooms, and facilitating video-based Q&A sessions between clinicians and patients, to identify nutritional gaps. Zhou et al. (36), meanwhile, made real-time adjustments to patients’ dry weight and dietary structures through online consultations and precision nutrition planning. Furthermore, Duan et al. (31) combined animated video education, face-to-face counseling, and WeChat-based supervision to enhance dietary adherence through phased interventions, while Li et al. (39) delivered personalized nutrition plans via web platforms and monitored patient activity levels, achieving diet management.

To understand how these interventions exert their effects, it is critical to examine key nutritional indicators. Biochemical indicators like hemoglobin, prealbumin, albumin, transferrin, calcium, potassium, and phosphorus are crucial for nutrition assessment in hemodialysis patients. The MQSGA is also a validated clinical nutrition evaluation tool (12, 45, 46). Overall, meta-analysis showed digital health technology-based nutrition interventions improved MQSGA, hemoglobin, albumin, prealbumin, phosphorus, and potassium but not transferrin and calcium. Specifically, for hemoglobin, while hemoglobin levels in hemodialysis patients are primarily influenced by erythropoietin (Epo) dosing, inflammatory status, and access-related blood loss, digital health interventions may provide supportive benefits. By offering personalized dietary guidance and promoting adherence to prescribed treatments, including medication and supplemental iron, digital tools may help optimize conditions for hemoglobin synthesis. It is important to note that intravenous iron is commonly used to manage iron deficiency in this population when indicated, and vitamin B12 deficiency is routinely screened for and is relatively uncommon. Thus, the role of digital interventions may lie mainly in enhancing overall treatment adherence and integrating nutritional support with medical management (47). Albumin and prealbumin: With the help of digital health technology, patients can more accurately control protein intake, avoid malnutrition caused by excessive restriction, and adjust dietary structure in time to ensure a sufficient high-quality protein supply, which is conducive to maintaining and improving serum albumin and prealbumin levels. Moreover, diet education helps patients understand protein metabolism, encouraging better adherence to diet plans. For phosphorus and potassium: Digital health technology monitors intake in real time and promptly reminds patients to adjust their diet within set safe ranges (48). For example, when the intake of phosphorus is close to the upper limit, the patient is reminded to reduce the intake of high-phosphorus foods, and at the same time, the patient is recommended to replace foods with low phosphorus and rich in other nutrients, so as to maintain the levels of phosphorus and potassium in a relatively stable range and avoid complications caused by abnormal phosphorus and potassium metabolism. However, transferrin is influenced by factors like inflammation, which is common in hemodialysis patients, potentially limiting improvement (49, 50). Meanwhile, calcium levels are regulated by complex mechanisms like PTH, vitamin D, and mineral metabolism, which may not be directly affected by nutritional interventions (51). Notably, sensitivity analysis showed unstable results for transferrin, which became significant after excluding a study by Wang LJ (34), indicating caution in interpreting results. More high-quality studies are needed. Subgroup analysis on phosphorus also showed no significant difference in the ≥ 6 months intervention subgroup or in mobile-platform-based interventions, inconsistent with the overall meta-analysis. Possible reasons include small sample sizes in subgroup analyses and reduced intervention effects due to declining patient compliance or adapted dietary habits over long-term interventions. Thus, future research should address compliance issues in long-term interventions, design 1- to 3-year follow-up studies, and conduct more large-sample, multicenter studies.

Beyond biochemical markers, anthropometric indicators provide complementary insights into nutritional status. In the context of anthropometric indicators, BMI is considered an independent predictor of mortality in MHD patients (52). Skinfold thickness measurement can be used to assess energy stored in the body in the form of fat, while mid-arm muscle circumference can reflect the retention of muscle protein (53). All of these are commonly used nutritional assessment indicators for hemodialysis patients. Nutrition interventions based on digital health technology can improve five outcome indicators during hemodialysis, namely the relative increase in body weight (%), weight gain, body mass index, mid-arm muscle circumference, and triceps skinfold thickness. Among these, for weight-related indicators, digital health technology can develop a personalized calorie intake plan for patients, combined with exercise guidance, to help patients control their body weight within a reasonable range. Regular weight monitoring and reminder functions enable patients to understand weight changes in a timely manner, adjust their diet and exercise habits, and thus effectively control weight gain. For BMI: By comprehensively considering the height, weight, and other information of patients, digital health technology can provide targeted diet and exercise advice to help patients maintain a healthy weight and body fat ratio, thereby improving BMI. For mid-arm muscle circumference and skinfold thickness: With the nutrition education function of digital health technology, patients can learn how to increase muscle protein intake through a reasonable diet, combined with appropriate resistance exercise, which can help increase muscle mass and improve mid-arm muscle circumference (54). At the same time, the precise control of fat intake can also help to regulate the distribution and thickness of subcutaneous fat and improve the skinfold thickness. However, for the outcome indicator of mid-arm muscle circumference, only four studies were included in the sensitivity analysis. The limited number of included studies resulted in a *p* ≥ 0.05, indicating poor stability of the results. Therefore, the study findings should be interpreted with caution. Future research should focus on conducting more high-quality studies to further explore this area.

For the secondary outcome of serum urea nitrogen levels, changes can reflect both the clearance efficiency of hemodialysis and the nutritional status of patients. In hemodialysis patients, the loss of renal function and decreased glomerular filtration rate, along with a chronic state of low-grade inflammation, can lead to elevated serum urea nitrogen levels. Serum creatinine is an important indicator of renal function, and in hemodialysis patients with compromised renal function, serum creatinine levels are often elevated. Specifically, for urea nitrogen, nutritional intervention reduces blood urea nitrogen production by improving the nutritional status of patients, increasing muscle protein synthesis, and reducing muscle breakdown (55). In addition, good nutritional support can enhance the patient’s immunity and overall body function, reduce the chronic inflammatory state, and also help to reduce urea nitrogen levels. Regarding serum creatinine, it is important to emphasize that serum creatinine levels are influenced by both muscle mass and dialysis clearance efficiency. Rational nutritional intake aims to improve the patient’s muscle mass and function. The reduction in serum creatinine observed in this study may be attributed primarily to the following mechanisms: diet management assisted by digital health technology optimizes fluid control and treatment compliance, significantly improving dialysis efficiency and enhancing creatinine removal; concurrently, nutritional support helps reduce pathological muscle breakdown, potentially moderating creatinine generation. Thus, the net effect is manifested as a decrease in serum creatinine levels (56). To further clarify the specific pathways by which DHT improves dialysis efficiency, its mechanisms can be broken down into three concrete aspects: (1) Real-time monitoring of nutritional intake (e.g., protein, fluids, potassium, and phosphorus) enables personalized dietary adjustments that prevent excessive inter-dialytic weight gain and solute accumulation, thus reducing the osmotic and volumetric burden during dialysis (57); (2) Medication and treatment reminders improve adherence to phosphate binders, erythropoiesis-stimulating agents, and dialysis sessions themselves, supporting more stable metabolic conditions (58); (3) Integration of serial biomarker trends (e.g., pre- and post-dialysis creatinine and urea reduction ratio) provides clinicians with actionable insights to adjust dialysis prescriptions, such as session duration, blood flow rate, or dialysate composition, enabling more precise and efficient solute clearance. It should be noted that serum creatinine is positively correlated with muscle mass (5). In healthy individuals or those with stable renal function, creatinine levels should correspond to muscle mass; low levels may indicate insufficient muscle tissue, while high levels may suggest impaired renal function (59). Therefore, the clinical goal is to maintain creatinine within a range that corresponds to adequate muscle mass and nutritional status, rather than simply reducing it. In this study, the overall meta-analysis results for serum urea nitrogen levels showed that nutrition interventions based on digital health technology can reduce both serum urea nitrogen and creatinine levels. However, the sensitivity analysis of these two outcomes included only three RCTs each. After sequentially excluding every one of these RCTs, the limited number of included studies resulted in poor stability of the results and no statistical significance. Therefore, more high-quality studies are still needed in the future to further validate these findings.

Notably, the core advantages of these digital health interventions lie in their dynamic adaptability, personalization, and accessibility: App-based algorithms enable real-time data analysis and instant feedback, social media platforms foster clinician-patient communication and behavioral monitoring, and remote technologies transcend spatiotemporal limitations of traditional care. However, their efficacy and widespread adoption are constrained by multiple practical and resource-related challenges. First, disparities in digital access and the complexity of digital solutions pose significant barriers. Since digital interventions rely on internet connectivity and smart devices, some patients, particularly those in resource-limited settings, may face obstacles in accessing such tools (60, 61). Additionally, developing specialized software or securing expert backend support demands substantial resources, complicating implementation (62, 63). Addressing these requires expanding digital infrastructure to ensure equitable access (64) and collaborating with information technology experts to design user-friendly applications with reliable systems, thereby minimizing technical barriers (65). Second, healthcare workforce constraints and workflow disruptions hinder adoption. Insufficient staff struggle to manage the extra workload introduced by digital interventions, such as data collection and follow-up (66, 67). Modifying workloads where feasible can help reduce disruptions and streamline integration. Third, broader economic, regulatory, and sociocultural factors impact viability. Excessively high costs for intervention groups (68), conflicts with local laws (65), and mismatches with cultural contexts (69) all impede uptake. Economic evaluations (e.g., cost–benefit analyses) can demonstrate long-term value, while adapting interventions to align with legal requirements and cultural norms is critical for acceptance. Looking ahead, future research should further leverage emerging technologies like AI-driven personalized recommendations and wearable biosensors, while exploring hybrid methodologies (e.g., gamification, family involvement) to enhance patient engagement. Prioritizing cultural adaptability, such as simplified interfaces for elderly users or multilingual support, will also be key to improving global applicability, alongside addressing the aforementioned barriers to ensure effective and equitable implementation.

## 5 Conclusion

The meta-analysis showed that digital health technology-based nutrition interventions can improve 13 indicators in hemodialysis patients: hemoglobin, albumin, prealbumin, phosphorus, potassium, MQSGA score, relative increase in body weight (%), weight gain, BMI, mid-arm muscle circumference, triceps skinfold thickness, blood urea nitrogen, and serum creatinine. However, no significant effects were found for transferrin and calcium. Due to the limited number of studies included for some outcomes and unstable results from sensitivity analyses and subgroup analyses, these findings should be interpreted with caution. Future research should focus on expanding the applications of digital health technologies, determining the optimal intervention methods, frequency, and duration, and conducting more large-scale, multicenter, high-quality randomized controlled trials to identify the best intervention protocols.

### 5.1 Limitations

(1) Due to the researchers’ language limitations, only Chinese and English studies were included. Moreover, most of the included studies were conducted in China, which indicates a geographical bias. Thus, the results of this study may have regional characteristics, and differences across regions need to be considered when generalizing the conclusions. (2) Among the 23 included studies, 4 were rated as low-quality, 3 as high-quality, and 16 as moderate-quality. The overall quality of the included studies was suboptimal, which might affect the stability of the results. (3) The studies were conducted in six countries, and differences in development, economy, and nursing environments may have influenced the findings. (4) Moreover, due to the limited number of included studies, subgroup analyses based on age and dialysis duration were not feasible. (5) Most included studies failed to adequately address or adjust for key baseline confounders (e.g., diabetes status and inflammatory markers), which significantly influence nutritional outcomes. This omission introduces a potential for confounding bias, meaning the observed effects may not be solely attributable to the digital interventions. (6) The analysis of some specific outcome measures may result in unstable estimates due to the small number of included studies.

## Data Availability

The original contributions presented in the study are included in the article/Supplementary material, further inquiries can be directed to the corresponding author.
