# Supplementary material for: Impact of digital health technology-based nutritional interventions on the nutritional status of hemodialysis patients: a systematic review and meta-analysis
Source: Front Nutr. 2025 Oct 29;12:1681161. doi: 10.3389/fnut.2025.1681161 (PMC12604986; doi:10.3389/fnut.2025.1681161)
Supplement: Supplementary file 2 [file Data_Sheet_1.docx]

**Supplementary material**

| **Content** | **Pages** |
| --- | --- |
| **Table S1** Search strategy. | 1-3 |
| **Figure S1** Risk of bias figure. | 4 |
| **Figure S2** Risk of bias summary. | 5 |
| **Figure S3-17** The results of the meta-analysis. | 6-12 |
| **Figure S18** Funnel plot of hemoglobin. | 12 |
| **Figure S19** Funnel plot of albumin. | 13 |
| **Figure S20** Funnel plot of phosphorus. | 13 |

**Table S1** Search strategy.

| Literature library | Search queries | Search results |
| --- | --- | --- |
| WanFang | 主题:(互联网 OR 移动 OR 微信 OR 人工智能 OR app OR 远程医疗 OR 电子健康 OR 虚拟现实 OR 虚拟仿真 OR 虚拟环境 OR VR OR 虚拟) and 主题:(血透 OR 血液透析 OR 维持性血液透析 OR MHD) and 主题:(饮食 OR 营养) | 529 |
| CNKI | (主题:互联网 + 移动 + 微信 + 人工智能 + app + 远程医疗 + 电子健康 + 虚拟现实 + 虚拟仿真 + 虚拟环境 + VR + 虚拟) AND (主题:血透 + 血液透析 + 维持性血液透析 + MHD) AND (主题:饮食 + 营养) | 89 |
| VIP | ((((((((题名或关键词=互联网 OR 题名或关键词=移动) OR 题名或关键词=微信) OR 题名或关键词=人工智能) OR 题名或关键词=app) OR 题名或关键词=远程医疗) OR 题名或关键词=电子健康) OR 题名或关键词=虚拟现实) OR 题名或关键词=虛拟仿真) OR 题名或关键词=虛拟环境) OR 题名或关键词=VR) OR 题名或关键词=虛拟) AND ((题名或关键词=血透 OR 题名或关键词=血液透析) OR 题名或关键词=维持性血液透析) OR 题名或关键词=MHD)) AND (题名或关键词=饮食 OR 题名或关键词=营养)) | 29 |
| CBM | #1 ("肾透析"[不加权:扩展]) OR ( "血透"[常用字段:智能] OR "血液透析"[常用字段:智能] OR "维持性血液透析"[常用字段:智能] OR "MHD"[常用字段:智能]) | 81745 |
|  | #2 ("饮食护理"[不加权:扩展]) OR ( "饮食"[常用字段:智能] OR "营养"[常用字段:智能]) | 376842 |
|  | #3 ("互联网干预"[不加权:扩展] OR "移动应用"[不加权:扩展] OR "人工智能"[不加权:扩展] OR "远程医学"[不加权:扩展] OR "虚拟现实"[不加权:扩展]) OR ( "互联网"[常用字段:智能] OR "移动"[常用字段:智能] OR "微信"[常用字段:智能] OR "app"[常用字段:智能] OR "远程医疗"[常用字段:智能] OR "电子健康"[常用字段:智能] OR "虚拟仿真"[常用字段:智能] OR "虚拟环境"[常用字段:智能] OR "VR"[常用字段:智能] OR "虚拟"[常用字段:智能]) | 155780 |
|  | #4=#1 AND #2 AND #3 | 92 |
| PubMed | #1**((((((hemodialysis[Title/Abstract]) OR (hemodialysis[MeSH Terms])) OR (haemodialysis[MeSH Terms])) OR (haemodialysis[Title/Abstract])) OR (mhd[Title/Abstract])) OR ("renal dialysis"[Title/Abstract])) OR ("renal dialysis"[MeSH Terms])** | 161189 |
|  | #2(((((((mobile applications[MeSH Terms]) OR (smartphone[MeSH Terms]) OR (wearable electronic devices [MeSH Terms]) OR (digital health[MeSH Terms]) OR (telemedicine[MeSH Terms]) OR (artificial intelligence[MeSH Terms]) OR (internet[MeSH Terms]) OR (mhealth[Title/Abstract]) OR (web[Title/Abstract]) OR (wechat[Title/Abstract]))) OR (virtual reality[MeSH Terms])) OR (virtual reality[Title/Abstract])) OR (VR[Title/Abstract])) OR (virtual environment[Title/Abstract])) OR (virtual simulation[Title/Abstract]) | 634254 |
|  | #3**(((((((nutrition[Title/Abstract]) OR (nutri*[Title/Abstract])) OR (supplement*[Title/Abstract])) OR (diet therapy[Title/Abstract])) OR (diet*[Title/Abstract])) OR (nutrition[MeSH Terms])) OR (dietary[MeSH Terms])) OR (dietary[Title/Abstract])** | 1628529 |
|  | #4= #1 AND #2 AND #3 | 146 |
| Web of Science | #1**(((TS=(hemodialysis)) OR TS=(haemodialysis)) OR TS=(MHD)) OR TS=("renal dialysis")** | 242835 |
|  | #2**(((((((((((((TS=(mobile applications)) OR TS=(smartphone)) OR TS=(wearable electronic devices)) OR TS=(digital health)) OR TS=(telemedicine)) OR TS=(artificial intelligence)) OR TS=(internet)) OR TS=(mhealth)) OR TS=(Web)) OR TS=(Wechat)) OR TS=(virtual reality)) OR TS=(VR)) OR TS=(virtual environment)) OR TS=(virtual simulation)** | 2114355 |
|  | #3**(((((TS=(nutrition)) OR TS=(nutri*)) OR TS=(supplement*)) OR TS=(diet therapy)) OR TS=(diet*))** | 4431315 |
|  | #4=#1 AND #2 AND #3 | 363 |
| Cochrane Library | #1**( hemodialysis ):ti,ab,kw OR ( haemodialysis ):ti,ab,kw OR ( MHD ):ti,ab,kw OR ( renal dialysis ):ti,ab,kw** | **20837** |
|  | #2**( mobile applications ):ti,ab,kw OR ( smartphone ):ti,ab,kw OR ( wearable electronic devices ):ti,ab,kw OR ( digital health ):ti,ab,kw OR ( telemedicine ):ti,ab,kw OR ( artificial intelligence ):ti,ab,kw OR ( internet ):ti,ab,kw OR ( mhealth ):ti,ab,kw OR ( Web ):ti,ab,kw OR ( Wechat ):ti,ab,kw OR ( virtual reality ):ti,ab,kw OR ( VR ):ti,ab,kw OR ( virtual environment ):ti,ab,kw OR ( virtual simulation ):ti,ab,kw** | **64045** |
|  | #3**( nutrition ):ti,ab,kw OR ( nutri* ):ti,ab,kw OR ( supplement* ):ti,ab,kw OR ( diet therapy ):ti,ab,kw OR ( diet* ):ti,ab,kw** | 205164 |
|  | #4=#1 AND #2 AND #3 | 72 |
| Embase | #1‘hemodialysis’:ti,ab,kw OR ‘haemodialysis’:ti,ab,kw OR ‘MHD’:ti,ab,kw OR ‘renal dialysis’:ti,ab,kw | 150900 |
|  | #2**‘mobile applications’:ti,ab,kw OR ‘smartphone’:ti,ab,kw OR ‘wearable electronic devices’:ti,ab,kw OR ‘digital health’:ti,ab,kw OR ‘telemedicine’:ti,ab,kw OR ‘artificial intelligence’:ti,ab,kw OR ‘internet’:ti,ab,kw OR ‘mhealth’:ti,ab,kw OR ‘Web’:ti,ab,kw OR ‘Wechat’:ti,ab,kw OR ‘virtual reality’:ti,ab,kw OR ‘VR’:ti,ab,kw OR ‘virtual environment’:ti,ab,kw OR ‘virtual simulation’:ti,ab,kw** | **555672** |
|  | #3**‘nutrition’:ti,ab,kw OR ‘nutri*’:ti,ab,kw OR ‘supplement*’:ti,ab,kw OR ‘diet therapy’:ti,ab,kw OR ‘diet*’:ti,ab,kw** | 1930826 |
|  | #4=#1 AND #2 AND #3 AND | 199 |
| CINAHL | #1MH hemodialysis OR MH haemodialysis OR MH MHD OR MH renal dialysis | 15410 |
|  | #2MH **mobile applications** OR MH **smartphone** OR MH **wearable electronic devices** OR MH **digital health** OR MH **telemedicine** OR MH **artificial intelligence OR MH internet OR MH mhealth OR MH Web OR MH Wechat OR MH virtual reality OR MH VR OR MH virtual environment OR MH virtual simulation** | 131916 |
|  | #3**MH** **nutrition OR MH nutri* OR MH supplement OR MH diet therapy OR MH diet*** | 219894 |
|  | #4=#1 AND #2 AND #3 AND | 31 |
| Scopus | #1 TITLE-ABS-KEY ( hemodialysis OR haemodialysis OR mhd OR "renal dialysis" | 248291 |
|  | #2 TITLE-ABS-KEY ("mobile applications" OR smartphone OR "wear able electronic devices" OR "digital health" OR telemedicine OR "artificial intelligence" OR internet OR mhealth OR web OR wechat OR "virual reality" OR vr OR "virtual environment" OR "virtual simulation" ) | 2564308 |
|  | #3 TITLE-ABS-KEY (nutrition OR nutri* OR supplement* OR "diet therapy" OR diet* ) | 3207361 |
|  | #4=#1 AND #2 AND #3 AND | 288 |


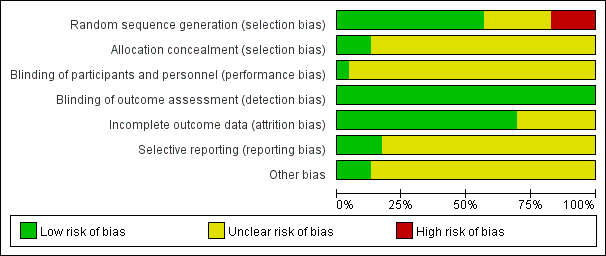


**Figure S1** Risk of bias figure.


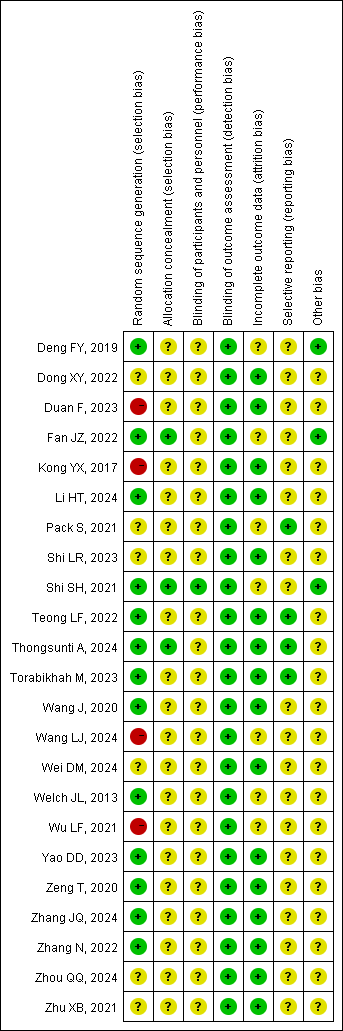


**Figure S2** Risk of bias summary.


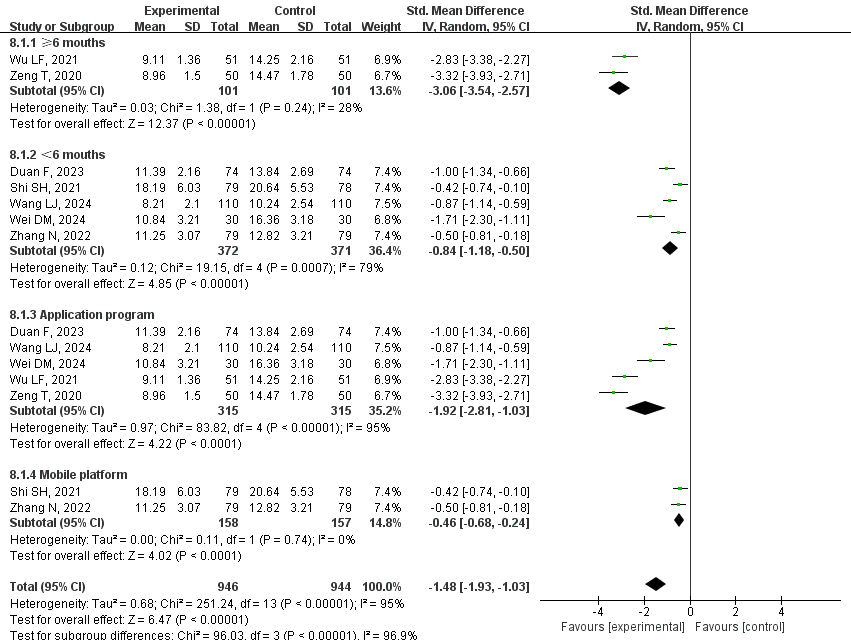


**Figure S3** Forest diagram of MQSGA.

Note: MQGSA, **Modified Quantitative Subjective Global Assessment;**


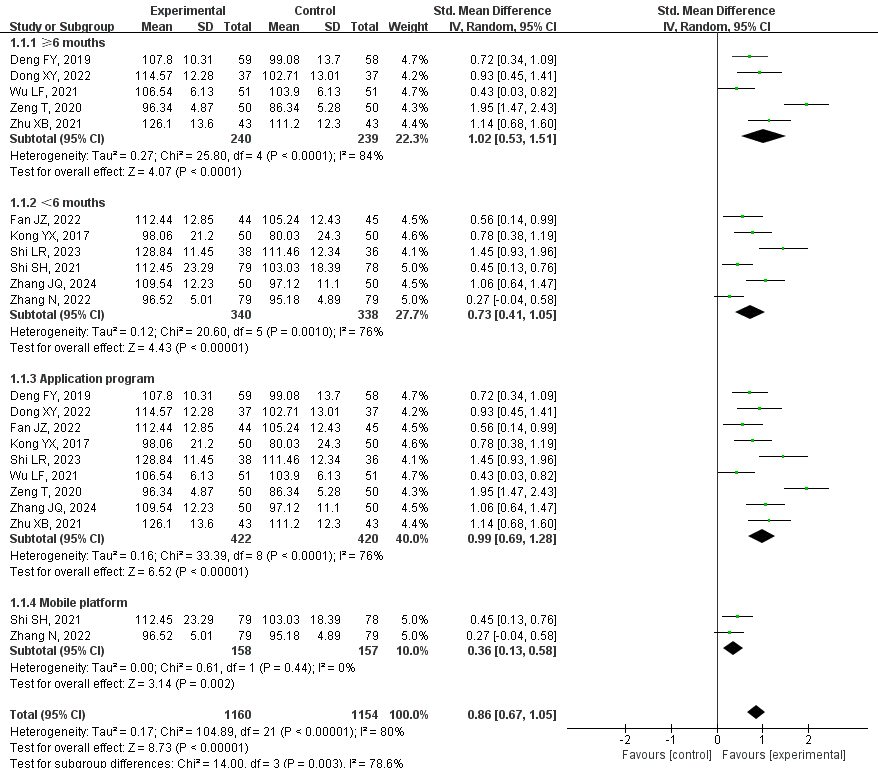


**Figure S4** Forest diagram of hemoglobin.


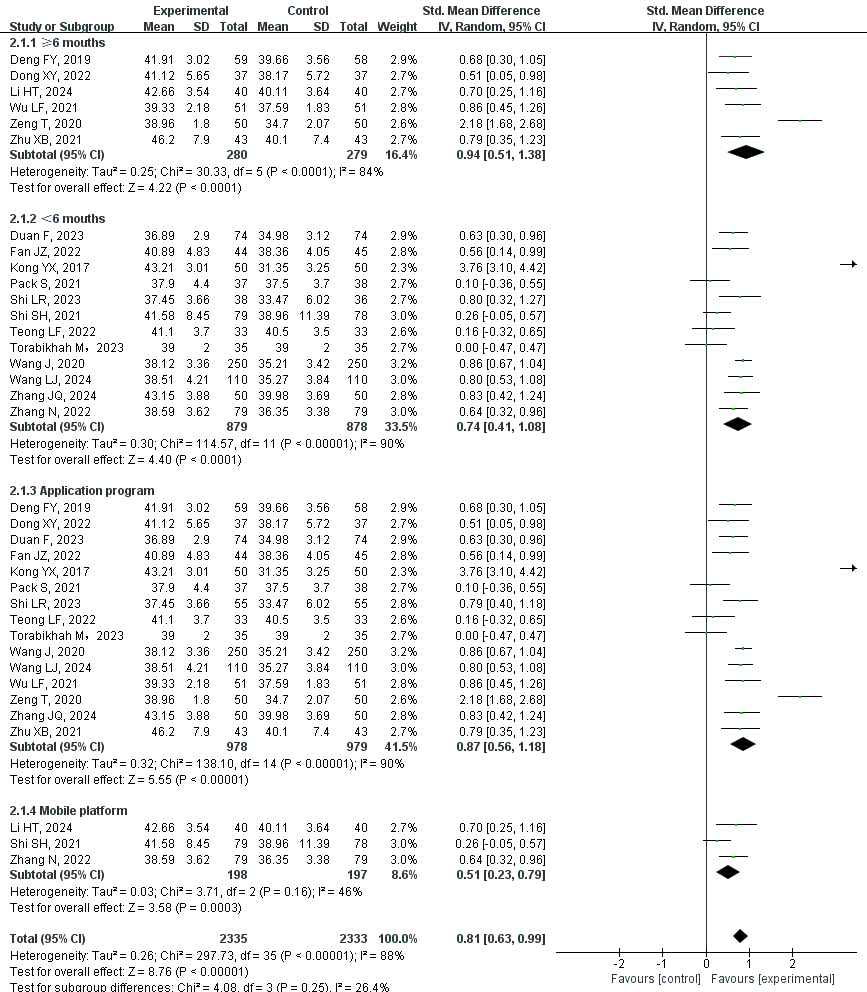


**Figure S5** Forest diagram of serum albumin.


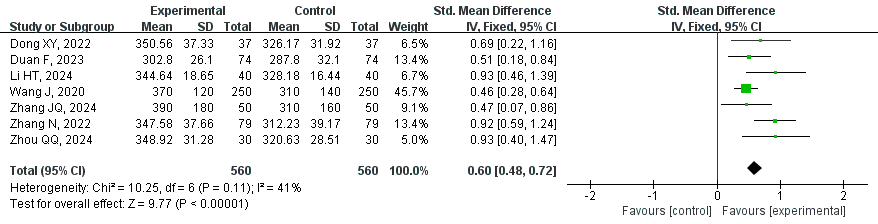


**Figure S6** Forest diagram of prealbumin.


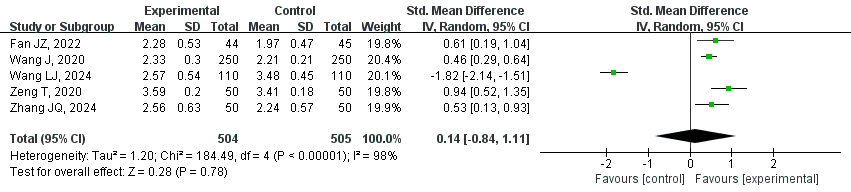


**Figure S7** Forest diagram of transferrin.


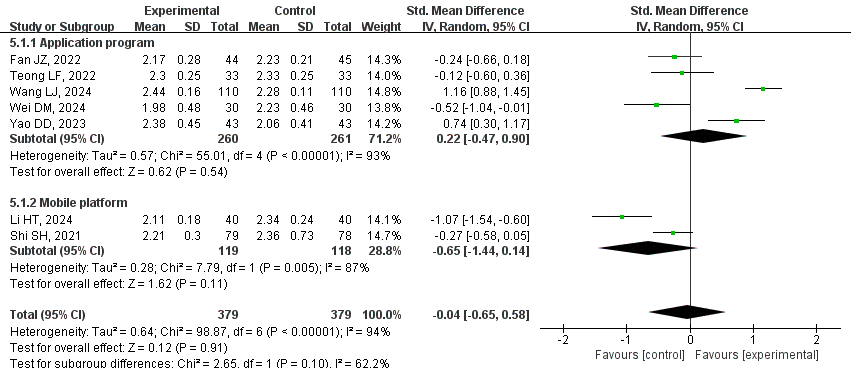


**Figure S8** Forest diagram of calcium.


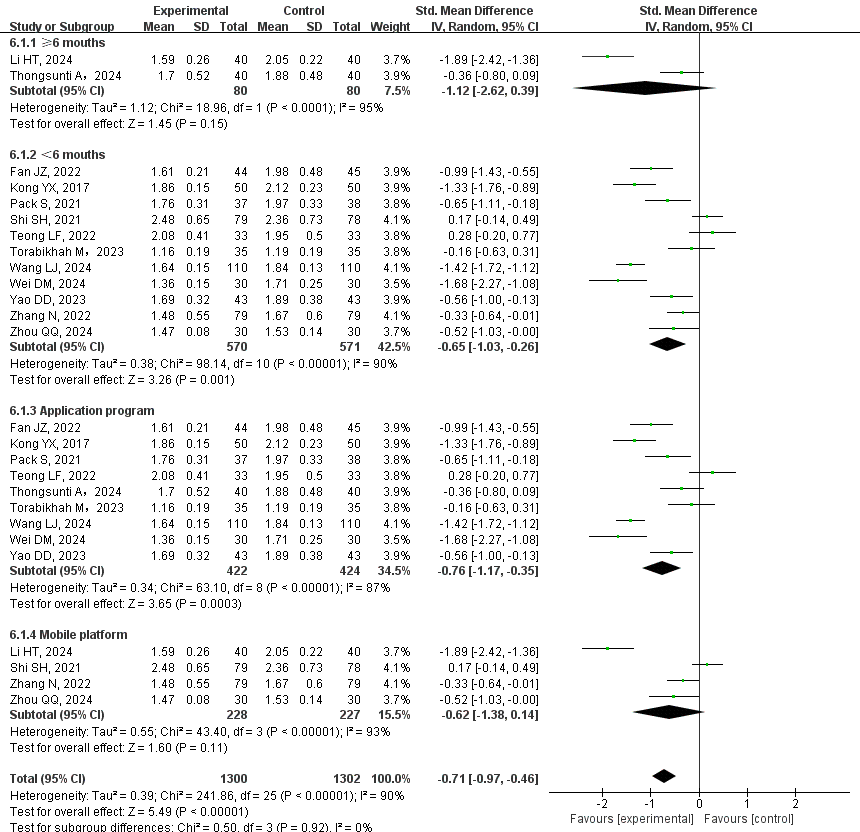


**Figure S9** Forest diagram of phosphorus.


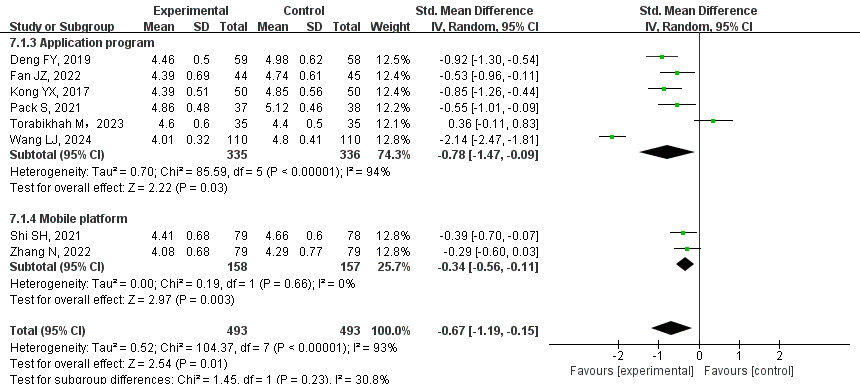


**Figure S10** Forest diagram of potassium.


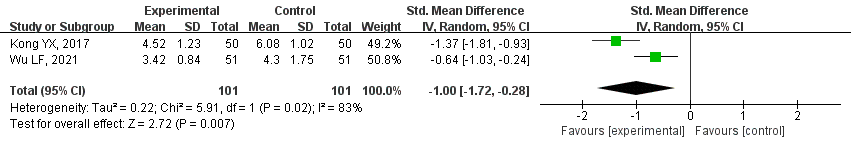


**Figure S11** Forest diagram of relative increase in body weight (%).


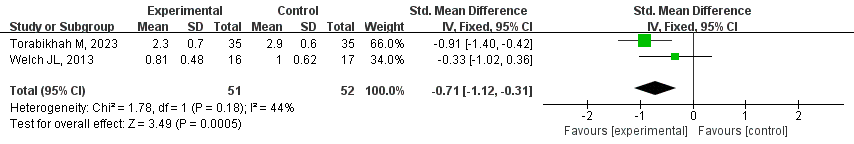


**Figure S12** Forest diagram of body weight gain.


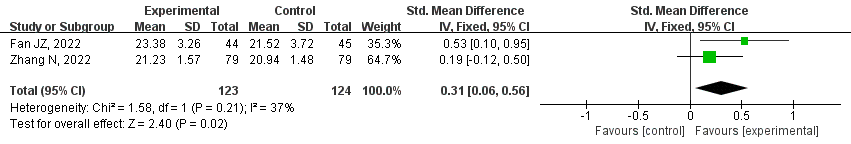


**Figure S13** Forest diagram of BMI.


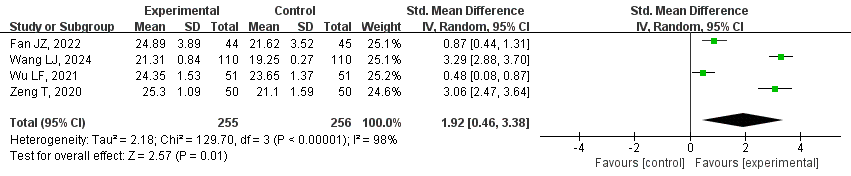


**Figure S14** Forest diagram of mid - arm muscle circumference.


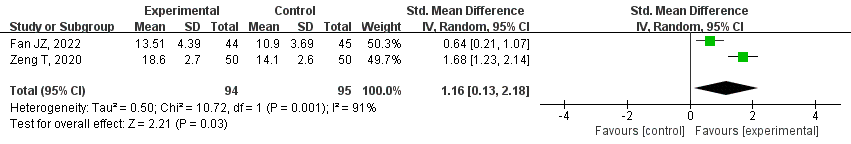


**Figure S15** Forest diagram of triceps skinfold thickness.


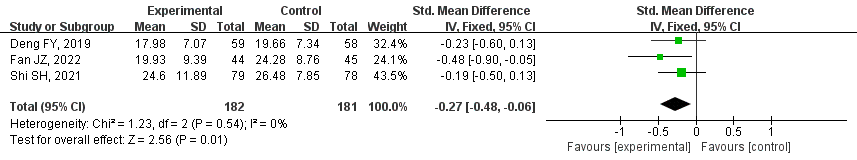


**Figure S16** Forest diagram of urea nitrogen.


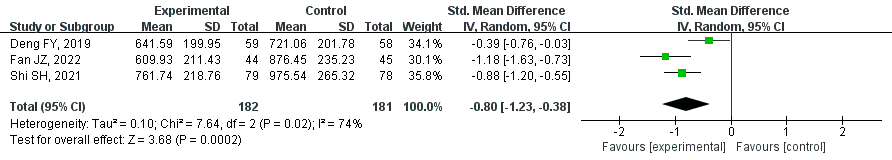


**Figure S17** Forest diagram of serum creatinine.


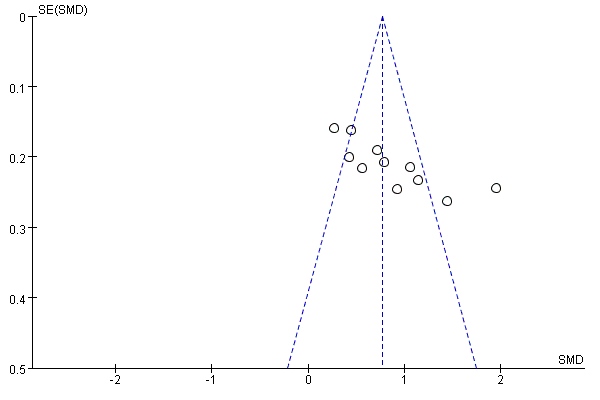


**Figure S18** Funnel plot of hemoglobin.


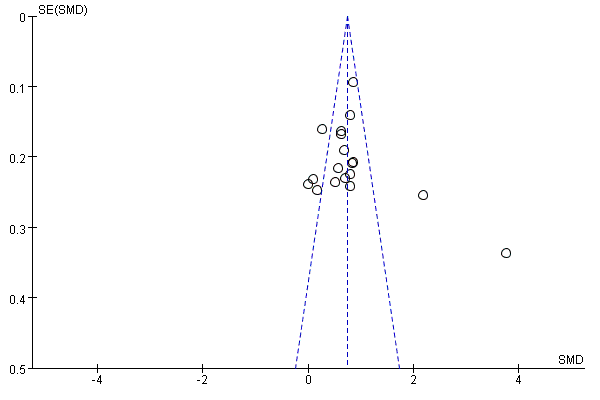


**Figure S19** Funnel plot of serum albumin.


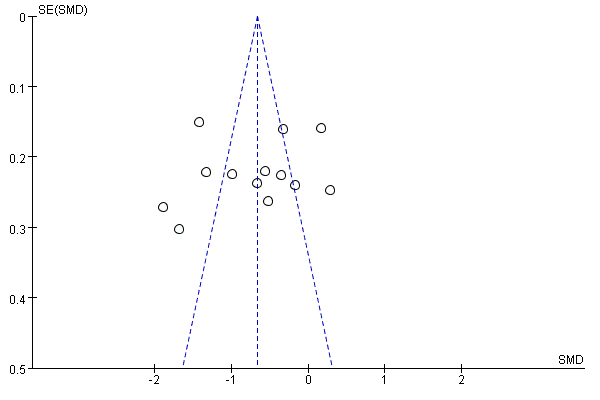


**Figure S20** Funnel plot of phosphorus.
